# Supplementary material for: Association of the Inferior Alveolar Nerve Position and Nerve Injury: A Systematic Review and Meta-Analysis
Source: Healthcare (Basel). 2022 Sep 16;10(9):1782. doi: 10.3390/healthcare10091782 (PMC9498832; doi:10.3390/healthcare10091782)
Supplement: Supplementary file 1 [file healthcare-10-01782-s001.zip › Table S1.pdf]

Table S1. Search strategy

Database	Search strategy
Cochrane Central Register of Controlled Trials (CENTRAL)	#1 MeSH descriptor: [Mandibular Nerve Injuries] explode all trees<br>#2 (Injury, Mandibular Nerve): ti,ab,kw<br>#3 (Mandibular Nerve Injury): ti,ab,kw<br>#4 (Nerve Injury, Mandibular): ti,ab,kw<br>#5 (Inferior Alveolar Nerve Injuries): ti,ab,kw<br>#6 (Lateral Pterygoid Nerve Injuries): ti,ab,kw<br>#7 (Masseteric Nerve Injuries): ti,ab,kw<br>#8 (Injury, Masseteric Nerve): ti,ab,kw<br>#9 (Auriculotemporal Nerve Injuries): ti,ab,kw<br>#10 (Auriculotemporal Nerve Injury): ti,ab,kw<br>#11 (Injury, Auriculotemporal Nerve): ti,ab,kw<br>#12 (Nerve Injuries, Auriculotemporal): ti,ab,kw<br>#13 (Nerve Injury, Auriculotemporal): ti,ab,kw<br>#14 (Deep Temporal Nerve Injuries): ti,ab,kw<br>#15 (Mental Nerve Injuries): ti,ab,kw<br>#16 (Injury, Mental Nerve): ti,ab,kw<br>#17 (Mental Nerve Injury): ti,ab,kw<br>#18 (Buccal Nerve Injuries): ti,ab,kw<br>#19 (Injury, Buccal Nerve): ti,ab,kw<br>#20 (Nerve Injury, Buccal): ti,ab,kw<br>#21 (Buccal Nerve Injury): ti,ab,kw<br>#22#1 OR #2 OR #3 OR #4 OR #5 OR #6 OR #7 OR #8 OR #9 OR #10 OR #11 OR #12 OR #13 OR #14 OR #15 OR #16 OR #17 OR #18 OR #19 OR #20 OR #21<br>#23(canal): ti,ab,kw<br>#24 #22 AND #23
Journals@Ovid	Mandibular Nerve Injuries or Injury, Mandibular Nerve or Mandibular Nerve Injury or Nerve Injury, Mandibular or Inferior Alveolar Nerve Injuries or Lateral Pterygoid Nerve Injuries and canal
web of science	TS = (Mandibular Nerve Injuries OR Injury, Mandibular Nerve OR Mandibular Nerve Injury OR Nerve Injury, Mandibular OR Inferior Alveolar Nerve Injuries) AND TS = canal
