# Supplementary material for: Association of the Inferior Alveolar Nerve Position and Nerve Injury: A Systematic Review and Meta-Analysis
Source: Healthcare (Basel). 2022 Sep 16;10(9):1782. doi: 10.3390/healthcare10091782 (PMC9498832; doi:10.3390/healthcare10091782)
Supplement: Supplementary file 1 [file healthcare-10-01782-s001.zip › Table S2.pdf]

Table S2. Results of quality assessment using the Newcastle-Ottawa Scale for a meta-analysis.

Study	Selection				Comparability	Outcome		
	Representativeness of the exposed cohort	Selection of the non-exposed cohort	Ascertainment of exposure	Demonstration that outcome of interest was not present at start of study	Comparability of cohorts on the basis of the design or analysis	Assessment of outcome	Was follow-up long enough for outcomes to occur	Adequacy of follow up of cohorts
Hasegawa,2013		×	×	×	×	×	×	×
Jun,2013	×	×	×	×	×			×
Nakayama,2009	×	×	×	×	×	×		×
Neves,2012	×	×	×	×	×	×		×
Tachinami,2017	×	×	×	×	××	×	×	×
Qi，2019		×	×	×	×	×	×	×
ueda,2012	×	×	×	×	×	×		×
shiratori,2013		×	×	×	×	×	×	

× means getting a star in this category, the comparability category can be awarded ××mostly.
