# Supplementary material for: Association of the Inferior Alveolar Nerve Position and Nerve Injury: A Systematic Review and Meta-Analysis
Source: Healthcare (Basel). 2022 Sep 16;10(9):1782. doi: 10.3390/healthcare10091782 (PMC9498832; doi:10.3390/healthcare10091782)
Supplement: Supplementary file 1 [file healthcare-10-01782-s001.zip › Table S3.pdf]

Table S3. Results of quality assessment using the Newcastle-Ottawa Scale for a meta-analysis.

	**Selection**				**Comparability**	**Exposure**		
Study	Is the case definition adequate	Representativeness of the cases	Selection of Controls	Definition of Controls	Comparability of cases and controls on the basis of the design or analysis	Ascertainment of exposure	Same method of ascertainment for cases and controls	Non-Response rate
Kubota, 2020		×		×	××	×	×	

× means getting a star in this category, the comparability category can be awarded ×× mostly.
